# Multimorbidity, polypharmacy, and mortality in older patients with pacemakers

**DOI:** 10.1002/joa3.12660

**Published:** 2021-11-23

**Authors:** Toshihiko Goto, Kento Mori, Takafumi Nakayama, Junki Yamamoto, Yasuhiro Shintani, Kazuaki Wakami, Hidekatsu Fukuta, Yoshihiro Seo, Nobuyuki Ohte

**Affiliations:** ^1^ Department of Cardiology Nagoya City University Graduate School of Medical Sciences Nagoya Japan; ^2^ Clinical Research Management Center Nagoya City University Hospital Nagoya Japan; ^3^ Department of Cardiovascular Medicine Nagoya City University East Medical Center Nagoya Japan

**Keywords:** mortality, multimorbidity, older patients, pacemakers, polypharmacy

## Abstract

**Background:**

The prevalence of multimorbidity and polypharmacy and its association with all‐cause mortality in older patients with pacemakers are largely unknown. We aimed to clarify the prevalence of multimorbidity and polypharmacy, and its association with all‐cause mortality in patients ≥75 years of age with pacemakers.

**Methods:**

We retrospectively investigated 256 patients aged ≥75 years (mean age 84.0 ± 5.3 years; 45.7% male) with newly implanted pacemakers. The study endpoint was all‐cause mortality (“with events”). Multimorbidity was defined as a Charlson Comorbidity Index ≥3. Polypharmacy was defined as the use of ≥5 medications.

**Results:**

During the follow‐up period (median, 3.1 years), 60 all‐cause deaths were reported. The Charlson Comorbidity Index (2.9 ± 1.9 vs. 1.7 ± 1.7, *p *< .001) and prevalence of multimorbidity (56.7% vs. 26.0%, *p *< .001) were significantly higher in deceased patients than in survivors. The number of drugs (6.9 ± 3.0 vs. 5.9 ± 3.3, *p *= .03) and the prevalence of polypharmacy (78.3% vs. 63.8%, *p *= .04) were significantly higher in patients with events than in those without events. The event‐free survival rate was significantly higher among patients without multimorbidity than in those with multimorbidity (log‐rank, *p *< .001), and was also significantly higher among patients without polypharmacy than in those with polypharmacy (log‐rank, *p *< .001). Multimorbidity (hazard ratio [HR]: 3.21; 95% confidence interval [CI]: 1.85–5.58; *p *< .001) and polypharmacy (HR: 1.97; 95% CI: 1.03–3.77; *p *= .04) were independent predictors of all‐cause mortality.

**Conclusions:**

Multimorbidity and its associated polypharmacy, which are common in the older population, are prevalent in patients with pacemakers and are independent predictors of poor prognosis.

## Graphical abstract

Multimorbidity and its associated polypharmacy are prevalent in older patients with pacemakers. Polypharmacy is commonly observed regardless of disease prevalence. Multimorbidity, polypharmacy, and dependent ADL were independent predictors of poor prognosis in such patients.

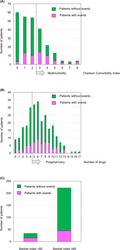

## 1 INTRODUCTION

With the increased number of older individuals in the Japanese population, patients with multimorbidity, defined as individuals living with two or more chronic health conditions, are often encountered.^1^ Multimorbidity is associated with decreased quality of life, impaired functional status, reduced physical and mental health, and increased mortality.^2^ Furthermore, multimorbidity is associated with a high treatment burden, polypharmacy, and considerably greater health service usage, including emergency hospital admission and a corresponding increase in medical expenditures.^3^ Simultaneously, patients who require a pacemaker (PM) implant are more often older than previously observed.^4^, ^5^ Therefore, multimorbidity is a major challenge for older patients with PMs. However, data on multimorbidity in older patients with PMs are scarce. Moreover, polypharmacy likely leads to worse outcomes.^6^ Nevertheless, data on polypharmacy in such patients are limited. In addition, aging leads to a decline in activities of daily living (ADL). Accordingly, this study aimed to clarify the prevalence of multimorbidity, polypharmacy, and dependent ADL, and to evaluate its association with all‐cause mortality in patients with PMs aged ≥75 years.

## 2 METHODS

### 2.1 Study population

This was a single‐center, retrospective, observational study. A total of 374 consecutive patients with newly implanted PMs for standard pacing indications were investigated at Nagoya City University Hospital between January 2010 and June 2020. We collected baseline characteristics, comprehensive echocardiographic indices, laboratory data, PM parameters (including pacing mode: physiological or ventricular pacing), underlying diseases, and the number of drugs taken. All data were extracted from the patients’ medical records. Blood samples, echocardiographic indices, underlying disease, data regarding the number of drugs taken, and Barthel index (BI)^7^ were collected at discharge. The individual percentages of right ventricular pacing were also obtained from the medical records as the mean value for each individual during the 3‐month period prior to the end of the study. The study endpoint was all‐cause mortality (“with events”). Patients aged ≥75 years were eligible for inclusion if they had been followed up for at least 6 months. Patients aged <75 years (*n* = 109) and those who had been followed up for less than 6 months (*n* = 9) were excluded. The study protocol was approved by the Nagoya City University Graduate School of Medical Sciences and Nagoya City University Hospital Institutional Review Board (reference no. 60‐20‐0210) and was carried out in accordance with the principles of the Declaration of Helsinki. The requirement for written informed consent was waived by the Nagoya City University Graduate School of Medicine and the University Hospital Institutional Review Board due to the retrospective nature of the study.

### 2.2 Multimorbidity, polypharmacy, and BI

The Charlson Comorbidity Index (CCI),^8^ which predicts mortality by classifying or weighting comorbidities, was measured, with the severity of comorbidities categorized as follows: mild, CCI scores of 1–2; moderate, scores of 3–4; and severe, scores ≥5. In this study, multimorbidity was defined as a CCI score of ≥3. The underlying disease definitions were derived from the CCI criteria. All regular daily medications at discharge were counted. Polypharmacy was defined as the daily use of ≥5 regular medications. The BI consists of 10 items: feeding, bathing, grooming, dressing, bowel control, bladder control, toileting, chair transfer, ambulation, and stair climbing. This index is widely used as an indicator of ADL in routine clinical practice in geriatric medicine (BI range, 0–100; BI ≥85 indicates independent ADL).^7^

### 2.3 Statistical analysis

The SPSS statistical software (version 23.0, SPSS Inc.) was used for all statistical analyses. Continuous variables are presented as means ± standard deviations for normally distributed variables, and as medians and interquartile ranges (IQR) for non‐normally distributed variables. Categorical variables are summarized as frequencies (%). For the comparison of two groups, continuous variables were compared using unpaired Student's *t*‐tests for normally distributed variables and Mann–Whitney U tests for non‐normally distributed variables. Differences in prevalence between the groups were compared using the Chi‐square test. For event‐free survival analysis, Kaplan–Meier curves were obtained and compared using log‐rank tests. We calculated hazard ratios (HRs) derived from the multivariate Cox regression analysis to identify predictors of all‐cause mortality. We used significant variables that were identified in univariate Cox regression models with a *p*‐value of <.05; these included the following: age, atrioventricular block (AVB) and sick sinus syndrome (SSS) as indications for PM implantation, multimorbidity, polypharmacy, and dependent ADL (BI <85). As there were too many individual factors related to underlying diseases and medications, and also because this study focused on the association between multimorbidity and the number of drugs and prognosis, the CCI and polypharmacy were used as representative variables. A *p*‐value <.05 was considered statistically significant.

## 3 RESULTS

### 3.1 Baseline characteristics

The final analysis included 256 patients (mean age 84.0 ± 5.3 years; 45.7% male). During the follow‐up period (median 3.1 [IQR, 1.5–5.2] years), 60 all‐cause deaths were reported. The causes of death were senility (*n* = 18), malignant disease (*n* = 14), infection (*n* = 8), renal failure (*n* = 3), intestinal hemorrhage (*n* = 1), and trauma (*n* = 1). Cardiovascular deaths, including heart failure (*n* = 8), sudden death (*n* = 5), and cerebral infarction (*n* = 2), were observed in 15 patients.

Table 1 lists the baseline characteristics of the patients. The age tended to be higher in patients with events defined as all‐cause death, rather than in those without events; however, this difference was not significant. Indications for PM implantation included SSS (*n* = 115), AVB (*n* = 132), and atrial fibrillation (AF) with bradycardia (*n* = 9). Case of AF and SSS and SSS alone were counted together. Of the 115 patients with SSS, 62 (53.9%) also had paroxysmal or persistent AF. One, 39, and 92 patients had a Mobitz type II AVB, advanced AVB, and complete AVB, respectively, with sensed QRS intervals of 118 ms, 107.4 ± 27.4 ms, and 117.8 ± 27.3 ms, respectively. The sensed QRS intervals for patients with SSS and AF with bradycardia were 97.9 ± 20.2 ms and 108.9 ± 30.5 ms, respectively. The paced QRS intervals for patients with SSS, Mobitz type Ⅱ AVB, advanced AVB, complete AVB, and AF with bradycardia were 145.0 ± 20.4 ms, 186 ms, 142.2 ± 19.6 ms, 150.3 ± 18.3 ms, and 137.0 ± 21.1 ms. The paced QRS intervals in patients with SSS were tended to be shorter than those of patients with AVB (145.0 ± 20.4 vs. 148.0 ± 19.3 ms, *p *= .23). Seventeen of 115 patients with SSS, 1 patient with Mobitz type II AVB, 17 of 39 patients with advanced AVB, 23 of 92 patients with complete AVB, and two of nine patients with AF bradycardia died. Compared to survivors, patients who were deceased had a significantly lower prevalence of SSS (28.3% vs. 50.0%, *p *= .003). The prevalence of SSS type 1 did not differ between the patients without events and those with events (4.6% vs. 8.3%, *p* = .27). The prevalence of SSS type 2 in patients without events was significantly higher than in those with events (18.4% vs. 5%, *p *= .01). The prevalence of SSS type 3 did not differ between the patients without events and those with events (27.0% vs. 15.0%, *p* = .06). Compared to the survivors, deceased patients had a significantly higher prevalence of AVB (68.3% vs. 46.4%, *p *= .003). The prevalence of Mobitz type II did not differ between the patients without events and those with event (0.0% vs. 1.7%, *p *= .07). The prevalence of advanced AV block in patients without events was significantly lower than in those with events (11.2% vs. 28.3%, *p *= .001). The prevalence of complete AV block did not differ between the patients without events and those with events (35.2% vs. 38.3%, *p *= .66). Two patients were found to have cardiac sarcoidosis at the time of PM implantation or during the follow‐up periods, and both survived without events. The PM settings for 94, 131, and 31 patients were DDD (R), DDD (R)/AAI (R), and VVI mode. Half of the patients had their PMs set to reduce ventricular pacing. Twenty‐three patients with the DDD (R) setting, 30 patients with the DDD (R)/AAI (R) setting, and 7 patients with the VVI mode PM setting died. The number of patients with a right ventricular pacing rate ≥40%, a threshold value for increased risk of heart failure,^9^ was significantly higher among those with events than among those without events, reflecting the indication for PM implantation (66.7% vs. 49.0%, *p *= .02). Among patients with DDD PMs for whom atrial data were available (*n* = 225), 64 patients had AF in the 3 months before the study ended for each patient, with a median AF burden of 10.0% (IQR 2.0%–79.3%).

**TABLE 1:** Comparison of the clinical characteristics

Characteristic	All patients	Without events^a^	With events^a^	*p* value
Number of patients (male %)	256 (45.7)	196 (43.4)	60 (53.3)	.18
Age (years)	84.0 ± 5.3	83.7 ± 5.1	85.1 ± 5.7	.07
Height (cm)	154.1 ± 9.6	154.5 ± 9.5	152.7 ± 9.9	.21
Weight (kg)	52.3 ± 10.5	52.8 ± 10.2	50.7 ± 11.4	.18
Body mass index (kg/m^2^)	21.8 ± 3.9	21.8 ± 3.9	21.6 ± 4.0	.77
LV ejection fraction (%)	67.4 ± 11.8	67.4 ± 11.5	67.1 ± 12.6	.84
Pacemaker parameters				
Sick sinus syndrome (%)	115 (44.9)	98 (50)	17 (28.3)	.003
Atrioventricular block (%)	132 (51.6)	91 (46.4)	41 (68.3)	.003
Atrial fibrillation (%)	9 (3.5)	7 (3.6)	2 (3.3)	.93
Pacing rate (bpm)	57.4 ± 7.1	57.0 ± 7.4	58.8 ± 6.2	.06
Paced QRS intervals (ms)	146.6 ± 19.8	146.7 ± 20.3	146.5 ± 18.5	.95
Sensed QRS intervals (ms)	106.9 ± 25.9	105.2 ± 25.7	112.5 ± 25.6	.06
Physiologic/ventricular pacing	225/31	172/24	53/7	.90
Ventricular pacing >40% (%)	53.1	49.0	66.7	.02
CCI score	2.0 ± 1.8	1.7 ± 1.7	2.9 ± 1.9	<.001
Multimorbidity (CCI score ≥3) (%)	33.2	26.0	56.7	<.001
Laboratory data				
BNP (mg/dl), median [interquartile range]	119.1 [66.1–298.1]	102.7 [63.8–239.9]	196.8 [74.8–397.0]	.02
HemoglobinA1c (%)	6.1 ± 0.7	6.1 ± 0.7	6.1 ± 0.8	.97
Serum creatinine (mg/dl)	1.2 ± 1.6	1.1 ± 1.0	1.5 ± 2.7	.20
eGFR (ml/min/1.73 m^2^)	57.4 ± 22.9	57.8 ± 21.2	56.0 ± 27.7	.61
Number of drugs	6.1 ± 3.3	5.9 ± 3.3	6.9 ± 3.0	.03
Polypharmacy (%)	67.2	63.8	78.3	.04
Barthel index	92.1 ± 21.3	93.8 ± 19.4	86.7 ± 26.2	.06
Barthel index <85 (%)	18.0	12.8	23.3	.046

Note.

Data are expressed as mean ± standard deviation or number or frequency (%).

Abbreviations: BNP, B‐type natriuretic peptide; CCI, Charlson Comorbidity Index; eGFR, estimated glomerular filtration rate; LV, left ventricular.

^a^ Events were defined as all‐cause death.

### 3.2 Multimorbidity, polypharmacy, and dependent ADL

**FIGURE 1:**
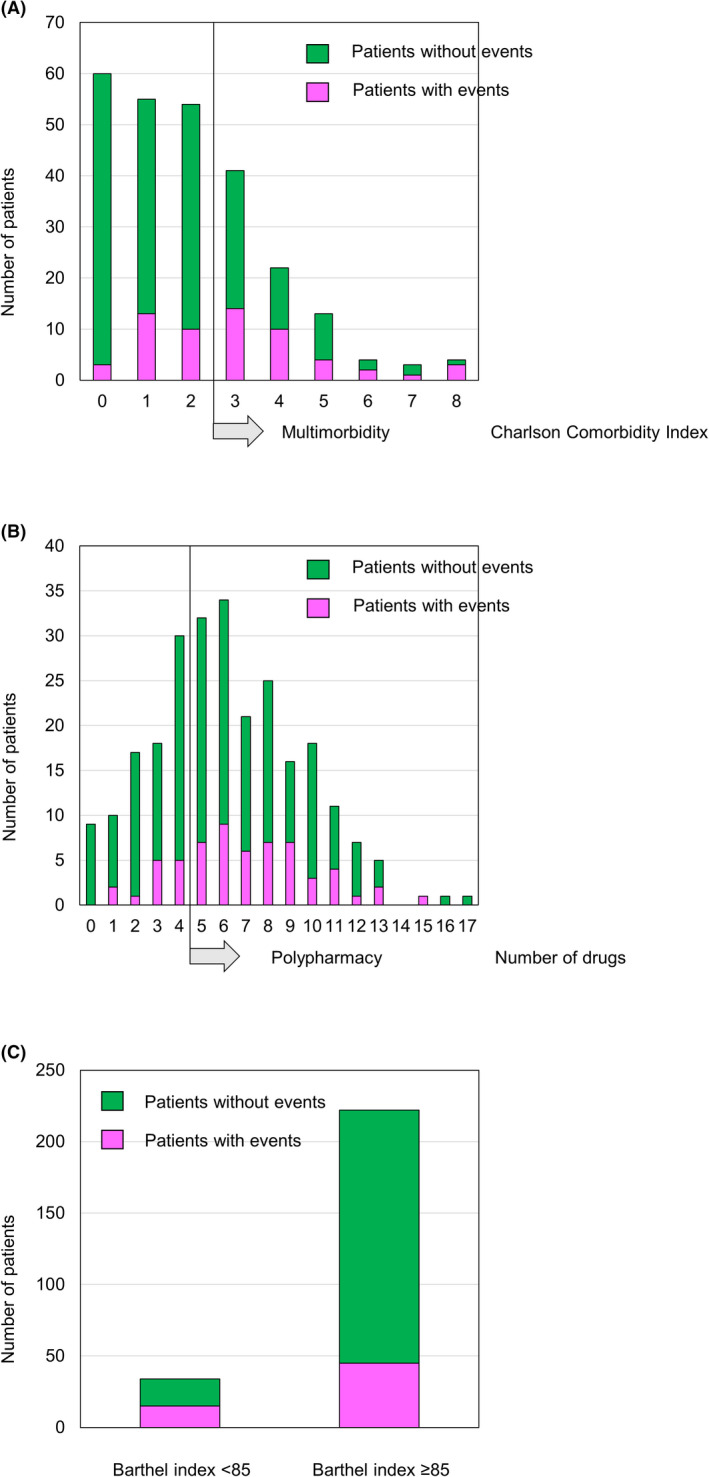
Distribution of (A) the Charlson Comorbidity Index (CCI), (B) the number of drugs, and a Barthel index value of 85 as a cutoff between patients who died of any cause (“with events”) and those who survived. The proportion of patients who died was higher among patients with multimorbidity, with polypharmacy, and a Barthel index of <85

The CCI (2.9 ± 1.9 vs. 1.7 ± 1.7, *p *< .001) and the prevalence of multimorbidity (56.7% vs. 26.0%, *p *< .001) were significantly higher in patients with events than in those without events. With regards to the components of the CCI, the prevalence of prior heart failure (65.0% vs. 34.7%, *p *< .001) and peripheral arterial disease (10.0% vs. 2.0%, *p *= .005) were significantly higher in patients with events than in those without events (Table 2). In this study, patients with previous heart failure were those who have been prescribed heart failure medication, including angiotensin‐converting enzyme inhibitors, angiotensin receptor blockers, spironolactone, loop diuretics, and beta‐blockers as medication for heart failure, not tachycardia. Reflecting the underlying diseases, plasma B‐type natriuretic peptide levels were significantly higher in patients with events than in those without events (196.8 [IQR, 74.8–397.0] vs. 102.7 [IQR, 63.8–239.9], *p *= .02). The number of drugs (6.9 ± 3.0 vs. 5.9 ± 3.3, *p *= .03) and the prevalence of polypharmacy (78.3% vs. 63.8%, *p *= .04) were significantly higher in patients with events than in those without events. The mean number of drugs used regularly in the total cohort was 6.1 ± 3.3, which exceeded the definition of polypharmacy in this study. Therefore, polypharmacy was observed in 67.2% of the cohort. Off‐label or contraindicated medications were not observed. Details of prescription medications with a prescription frequency of ≥1% in all patients are shown in Table 3. Reflecting the underlying disease, the use of loop diuretics (36.7% vs. 23.5%, *p *= .04) and antiplatelet medication (45.0% vs. 26.0%, *p *= .005) were significantly higher in patients who died than in those who survived. The number of tablets per patient was 1.84 for anti‐diabetic drugs; 1.67 for antidepressants; 1.50 for antibiotics; 1.49 for laxatives; 1.38 for antivertigo medicines; and 1.33 for antiplatelet, urological agents, and Chinese medicine (Table 3). The BI at the time of discharge tended to be lower in patients who subsequently died than in survivors (86.7 ± 26.2 vs. 93.8 ± 19.4, *p *= .06); however, the difference was not significant. The prevalence of patients with dependent ADL was significantly higher in deceased patients than in survivors (23.3% vs. 12.8%, *p *= .046) (Table 1). Figure 1 shows the distribution of the CCI (Figure 1A), the number of drugs prescribed (Figure 1B), and the use of BI of 85 as the cutoff value (Figure 1C). The proportion of patients who died was higher for those with multimorbidity (40.0% vs. 15.2%, *p *< .001), polypharmacy (29.4% vs. 11.6%, *p *= .002), and dependent ADL (35.9% vs. 21.5%, *p *= .046) than for those without these factors.

**TABLE 2:** Comparison of the underlying disease of the patients

Underlying disease	All patients	Without events^a^	With events^a^	*p* value
Hypertension (%)	71.1	70.9	71.7	.91
DM (%)	21.1	19.4	26.7	.23
DM with organ damage (%)	9.8	8.2	15.0	.12
Prior history of MI (%)	9.8	8.2	15.0	.12
Heart failure (%)	41.8	34.7	65.0	<.001
Prior history of stroke (%)	15.2	13.8	20.0	.24
Hemiplegia (%)	2.3	1.5	5.0	.12
Dementia or Alzheimer's disease (%)	18.0	15.3	26.7	.05
Peripheral arterial disease (%)	4.0	2.0	10.0	.005
Pulmonary disease/asthma (%)	6.6	5.1	11.7	.07
Moderate/severe renal disease (%)	2.0	2.0	1.7	.86
Rheumatic or CTD (%)	1.6	1.5	1.7	.94
Gastric or peptic ulcer (%)	2.7	2.6	3.3	.75
Chronic liver disease (%)	3.9	3.6	5.0	.62
Moderate/severe liver disease (%)	0.7	0.5	1.7	.37
Solid cancer (%)	20.7	19.4	25.0	.35
Lymphoma (%)	2.0	1.5	3.3	.38

Note.

Data are expressed as frequency (%).

Abbreviations: CTD, connective tissue disease; DM, diabetes mellitus; MI, myocardial infarction.

^a^ Events were defined as all‐cause death.

**TABLE 3:** Comparison of the medications prescribed to the patients

Medications	All patients	Without events^a^	With events^a^	*p* value	Number of tablets per patients
Loop diuretics (%)	26.6	23.5	36.7	.04	1.01
Thiazide diuretics (%)	10.5	11.2	8.3	.52	1
Tolvaptan (%)	1.2	0.5	3.3	.08	1
Antiplatelet (%)	30.5	26.0	45.0	.005	1.33
Anticoagulation (%)	29.3	32.1	20.0	.07	1
Statins (%)	34.0	34.2	33.3	.90	1
Other lipid‐lowering drugs (%)	1.2	1.5	0	.34	1
ACEIs (%)	8.2	8.2	8.3	.97	1
ARBs (%)	46.1	48.0	40.0	.28	1.01
β‐blockers (%)	23.4	23.5	23.3	.98	1
Calcium channel blockers (%)	52.3	51.5	55.0	.64	1.01
Digitalis (%)	0.8	0.5	1.7	.37	1
Other antiarrhythmic drugs (%)	4.3	4.6	3.3	.67	1
Nitric acid preparations (%)	4.3	3.6	6.7	.30	1.09
Anti‐diabetic drugs (%)	17.6	16.8	20.0	.57	1.84
Insulin (%)	1.6	1.5	1.7	.94	1
Histamine H2‐receptor antagonists (%)	7.4	7.7	6.7	.80	1
Proton pump inhibitors (%)	45.7	43.4	53.3	.18	1
Other digestive medicine (%)	12.5	10.7	18.3	.12	1.03
Laxatives (%)	25.4	26.5	21.7	.45	1.49
Probiotics (%)	4.3	4.6	3.3	.67	1
Xanthine oxidase inhibitors (%)	15.6	13.3	23.3	.06	1
Antipyretic analgesics (%)	12.9	11.2	18.3	.15	1.18
Benzodiazepines (%)	9.8	8.2	15.0	.12	1
Non benzodiazepines (%)	11.7	10.7	15.0	.37	1
Anticonvulsants (%)	2.3	2.6	1.7	.69	1
Antiparkinsonian drugs (%)	1.6	1.0	3.3	.21	1.25
Antidepressants (%)	1.2	1.0	1.7	.68	1.67
Antitussive expectorant drugs (%)	4.3	4.1	5.0	.76	1.17
Bronchodilators (%)	3.5	3.6	3.3	.93	1.11
Antivertigo Medicines (%)	3.1	3.1	3.3	.92	1.38
Anti‐hyperkalemic agents (%)	3.1	2.6	5.0	.34	1
Steroids (%)	2.0	1.0	5.0	.051	1
Osteoporosis Medications (%)	3.1	2.6	5.0	.34	1
Vitamin‐ preparations (%)	15.6	16.3	13.3	.58	1.30
Urological agents (%)	11.7	12.2	10.0	.64	1.33
Antibiotics (%)	2.3	2.0	3.3	.56	1.5
Chinese medicine (%)	3.1	2.6	5.0	.34	1.33

Note.

Data are expressed as frequency (%).

Abbreviations: ACEI, angiotensin‐converting enzyme inhibitor; ARB, angiotensin receptor blocker.

^a^ Events were defined as all‐cause death.

**FIGURE 2:**
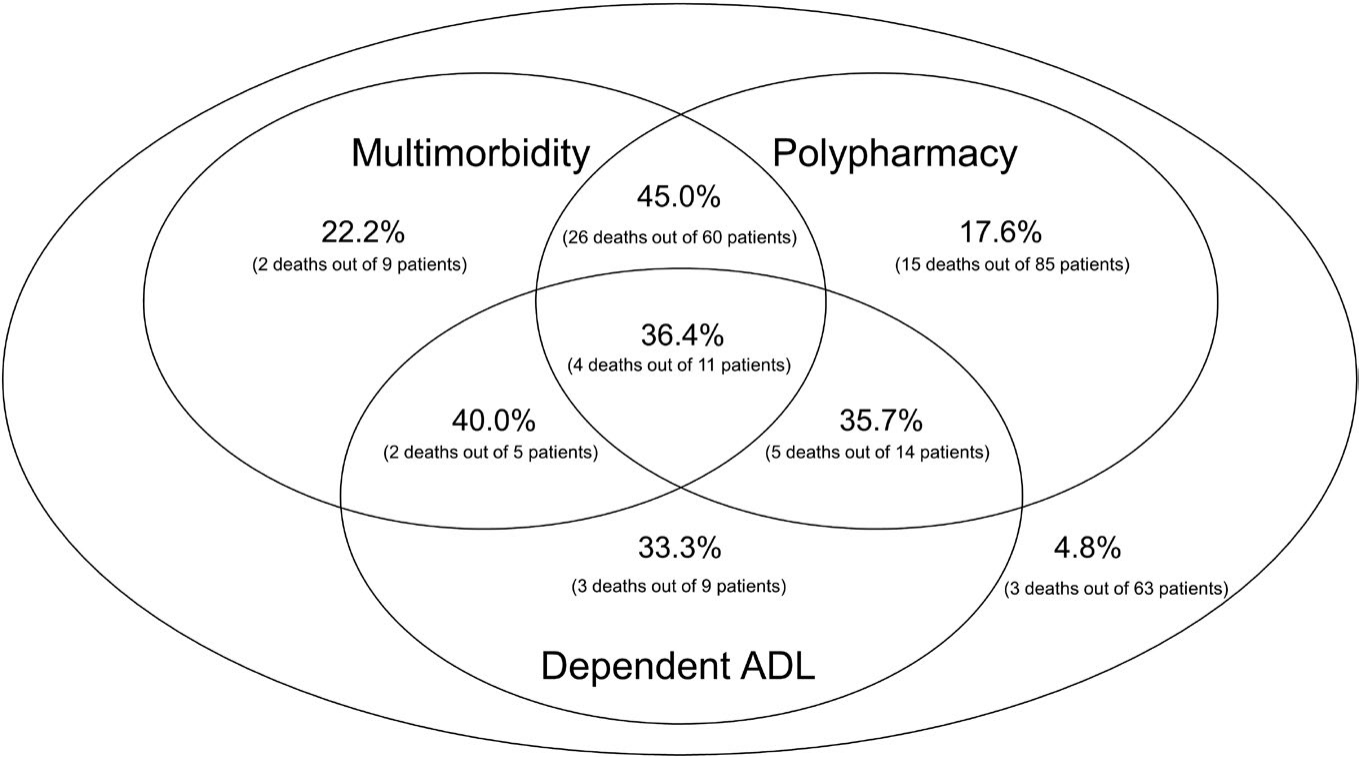
Venn diagram displaying the extent of overlap of multimorbidity with polypharmacy and dependent activities of daily living (ADL). The percentages of patients who died in each subgroup are expressed as frequencies (%). The number of each subgroup is indicated in parentheses. Total represented: 256 patients who had multimorbidity and/or polypharmacy and/or dependent ADL. Multimorbidity: overall *n* = 85 with the Charlson Comorbidity Index ≥3. Of these, 16 patients also had dependent ADL. Polypharmacy: overall *n* = 170 with the use of >5 medications; of these, 25 patients had dependent ADL. Dependent ADL: overall *n* = 39 with Barthel Index <85. Among patients with multimorbidity, polypharmacy, and dependent ADL, the proportion of patients with events was more than 35% among those with two or more of the three, and 4.8% among those with none of the three. An unexpectedly high number of patients with polypharmacy were present, including in the group without multimorbidity

Figure 2 shows a Venn diagram displaying the extent of overlap of multimorbidity with polypharmacy and dependent ADL. The proportion of patients with events was >35% of those with two or more of the three factors (multimorbidity, polypharmacy, and dependent ADL) and was 4.8% of those with none of the three. Among those who died, the proportion of patients with dependent ADL alone (33.3%) was higher than that for those with multimorbidity alone (22.2%) or polypharmacy alone (17.6%). Patients with multimorbidity were more likely to take multiple medications (83.5%; 71 out of 85 patients). Furthermore, even among those without multimorbidity, more than half of the patients received polypharmacy (57.9%; 99 of 171 patients).

### 3.3 Clinical outcome

**FIGURE 3:**
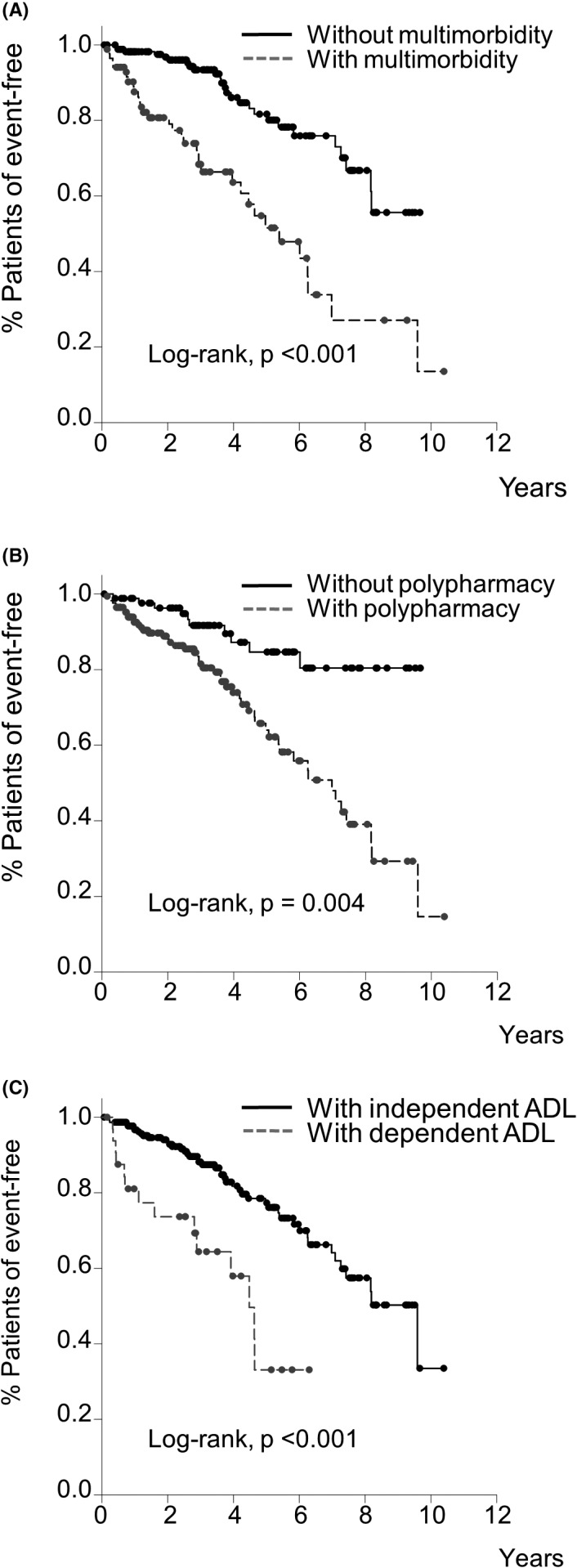
Kaplan–Meier curves for all‐cause mortality in patients with or without multimorbidity (A), with or without polypharmacy (B), and with dependent or independent ADL. The endpoint‐free survival rate was significantly higher in patients without multimorbidity and polypharmacy and with independent ADL than in those with multimorbidity, polypharmacy, and dependent ADL

The endpoint‐free survival rate was significantly higher in patients without multimorbidity (log‐rank, *p *< .001, Figure 3A), without polypharmacy (log‐rank, *p *= .004, Figure 3B), and with independent ADL (log‐rank, *p *< .001, Figure 3C) than in those with multimorbidity, polypharmacy, and dependent ADL.

In a multivariate Cox regression analysis, multimorbidity (HR: 3.21; 95% confidence interval [CI]: 1.85–5.58; *p *< .001), polypharmacy (HR: 1.97; 95% CI: 1.03–3.77; *p *= .04), and dependent ADL (HR: 2.74; 95% CI: 1.42–5.27; *p *= .003), as well as age (HR: 1.09; 95% CI: 1.03–1.14; *p *= .001) were identified as endpoint predictors during the follow‐up (Table 4). Paced QRS intervals, sensed QRS intervals, and LVEF were not considered as prognostic predictors. Although a higher prevalence of SSS type 2 and a lower prevalence of advanced AV block were observed in patients without events than in those with events, SSS and AVB as indications for PM implantation were not considered as prognostic predictors.

**TABLE 4:** Univariate and multivariate Cox regression analyses to identify predictors of all‐cause mortality

	Univariate				Multivariate			
	*β*	HR	95% CI	*p* value	*β*	HR	95% CI	*p* value
Age (years)	0.08	1.08	1.03–1.14	.002	0.09	1.09	1.03–1.14	.001
Male gender	0.46	1.58	0.95–2.63	.08				
Indication for PM implant								
AVB	0.69	1.99	1.15–3.43	.01				
SSS	−0.75	0.47	0.27–0.83	.01				
Atrial fibrillation	0.34	1.40	0.34–5.76	.64				
Multimorbidity	1.25	3.50	2.09–5.87	<.001	1.17	3.21	1.85–5.58	<.001
Polypharmacy	1.24	3.44	1.74–6.80	<.001	0.68	1.97	1.03–3.77	.04
Dependent ADL	1.20	3.33	1.79–6.18	<.001	1.01	2.74	1.42–5.27	.003

Abbreviations: ADL, activities of daily living; AVB, atrioventricular block;CI, confidence interval; HR, hazard ratio; PM, pacemaker; SSS, sick sinus syndrome.

## 4 DISCUSSION

Multimorbidity and the associated polypharmacy are common in older populations. In addition, aging leads to a decline in ADL. This study clarified the prevalence of multimorbidity, polypharmacy, and dependent ADL among older patients with a PM and demonstrated that these were independent predictors of all‐cause mortality among such patients. To the best of our knowledge, this is the first study to present a detailed description of multimorbidity, polypharmacy, and dependent ADL, and the factors that influence all‐cause deaths in older patients with PMs.

Multimorbidity is common in older patients with PMs and is an emerging problem in aging societies, in general. Diagnostic criteria for multimorbidity are not yet established. Although the National Institute for Health and Care Excellence (NICE) defined multimorbidity as a health condition in which patients live with two or more diseases,^10^ an internationally accepted definition has not been established. Therefore, different definitions have been used in separate studies, resulting in differing reported prevalence of multimorbidity. Prazeres and Santiago reported a significant difference in the prevalence of multimorbidity in the same patient cohort using two different definitions.^11^ Thus, a standardized definition of multimorbidity is required. In this study, CCI was used to express the number of diseases. A systematic review analyzing the pattern of multimorbidity suggested a list of diseases that should be included in the definition of multimorbidity.^12^ Among the top 10 suggested diseases (chronic obstructive pulmonary disease, diabetes, hypertension, malignancy, stroke, dementia, depression, joint disease, anxiety, and congestive heart failure), six diseases (diabetes, solid cancer, prior history of stroke, dementia or Alzheimer's disease, rheumatic or connective tissue disease, and heart failure) overlapped with CCI components. Five diseases (diabetes, hypertension, malignancy, stroke, and dementia) were the most frequent underlying diseases in this study. Therefore, we believe that the use of the CCI is appropriate. The prevalence of multimorbidity, defined as a CCI ≥3, was 33.2% in our study. A previous study reported that approximately 20% of patients over 75 years of age had a CCI ≥3.^13^ The differences observed are a result of the targeted cohorts; our study focused on patients with PMs, whereas the previous study focused on the general population. Since multimorbidity reflects an aging society, an extension of healthy life expectancy by improved lifestyles and/or social environment may help palliate this problem.

A previous study reported that cancer and vascular deaths accounted for approximately 80% of deaths in patients with multimorbidity.^14^ In the present study, approximately 50% of all deaths were caused by these two disease groups, while approximately 30% of deaths were due to senility. This difference results from a difference in cohorts; our study targeted much older patients than the aforementioned review. Therefore, the results of our study are consistent with the finding that cancer and cardiovascular deaths are the most common causes of death in patients with multimorbidity. Regarding the underlying diseases, the prevalence of prior heart failure was significantly higher in deceased patients than in survivors, and heart failure was one of the major causes of death in this study. However, although patients frequently died of malignant diseases, the prevalence of underlying malignant disease did not differ between the groups. Therefore, these underlying diseases did not directly lead to death. The so‐called “competing causes of death” were also observed in older patients with PMs.

This study showed that polypharmacy was common among older patients with PMs, with or without multimorbidity. One of the reasons for polypharmacy in patients without multimorbidity was that a single patient had multiple prescriptions for a single disease. Multiple medications were required for a single patient with diabetes, depression, and urological diseases in this study. Furthermore, multiple prescriptions were given to a single patient for diseases, such as constipation and vertigo. It has been suggested that polypharmacy has several adverse effects, including increased incidence of falls,^15^ frailty,^16^ fractures,^17^ renal dysfunction,^18^ and hospitalization.^19^ Polypharmacy likely leads to worse outcomes, given the higher likelihood of drug‐drug interactions and reduced treatment adherence.^6^ In particular, polypharmacy in patients with AF aged >75 years was associated with adverse outcomes.^20^ However, the exact definition of polypharmacy has not yet been established. A systematic review reported that the most common definition of polypharmacy was the concurrent use of five or more drugs,^21^ which is consistent with that used in our study. A previous study using this definition reported that the prevalence of polypharmacy was >15% in the general population of the United States.^22^ In contrast, Kojima et al. reported that more than half of geriatric outpatients were receiving six or more drugs.^15^

The NICE guidelines recommend a reduction in the number of medications and outline specific approaches to reduce the number of drugs,^10^ which creates a major treatment burden for patients with multimorbidity. However, the recommended guidelines are currently not supported by evidence from interventional studies. A systematic review did not reach definite conclusions regarding the efficacy of medication interventions for patients with multimorbidity; this was likely due to the small number of randomized clinical trials conducted in this area to date and the overall mixed findings.^23^

Polypharmacy is common in older patients, regardless of the number of diseases, as shown in this study. Polypharmacy leads to a treatment burden and increased overall national medical expenditures.^24^ Therefore, polypharmacy is a potential interventional target in daily clinical practice. However, the impacts of reducing the number of drugs may be different depending on the disease that causes polypharmacy. Reducing the number of anti‐diabetic drugs is not equal to reducing the number of drugs used for constipation. Recently, it was reported that discontinuing statins while maintaining other medications was associated with an increase in the long‐term risk of fatal and nonfatal cardiovascular outcomes in a cohort of older patients receiving polypharmacy.^25^ This result indicates that for some drugs, the disadvantages of discontinuation outweigh the benefits. On the other hand, discontinuation of some drugs may not pose a significant increase in risk. Therefore, prospective studies need to be conducted to determine those drugs and their disease groups that are less likely to be affected by reducing medications. Furthermore, safe methods of reducing medications must also be established. Appropriate intervention for polypharmacy may lead to improved patient outcomes and a reduced medical expenditure burden on society.

In this study, dependent ADL was found to be a predictor of all‐cause mortality. Patients with dependent ADL, defined as BI <85, had high mortality, even without multimorbidity or polypharmacy. This reflects the feature of BI: if ADL is maintained above a certain level, the BI can easily have a high score. Therefore, a BI <85 correlates with a considerable decline in ADL for the patient, and understandably, patients’ prognosis is poor even if BI <85 is the only factor.

This study has some limitations. First, this was a single‐center retrospective observational study that included a limited number of patients. Second, the numbers of diseases and medications were evaluated only at discharge. Since the number of diseases and drugs could change during the follow‐up period, our study could not clarify the impact of these changes on patients’ prognosis. Despite these limitations, we believe that the results of our study are meaningful. Finally, this study focused on whether three factors, multimorbidity, polypharmacy, and dependent ADL, affect the prognosis in patients with a PM. Therefore, no conclusions could be drawn regarding the impact of improvement of ADL by PM implantation on the prognosis. A further study is therefore needed to examine this issue.

## 5 CONCLUSIONS

Multimorbidity and its associated polypharmacy, which are common in older populations, are also prevalent in patients with PMs. Polypharmacy is commonly observed regardless of disease prevalence. Additionally, multimorbidity, polypharmacy, and dependent ADL were found to be independent predictors of poor prognosis, including among older patients with PMs.

## CONFLICTS OF INTEREST

Authors declare no conflict of interest for this article.

## Data Availability

All the relevant data are within the manuscript.

## References

[1] Salisbury C , Johnson L , Purdy S , Valderas JM , Montgomery AA . Epidemiology and impact of multimorbidity in primary care: a retrospective cohort study. Br J Gen Pract. 2011;61(582):e12–21. 21401985 10.3399/bjgp11X548929 PMC3020068

[2] National Guideline Centre (UK) . Multimorbidity: assessment, prioritisation and management of care for people with commonly occurring multimorbidity. London: National Institute for Health and Care Excellence (UK); 2016. 27683922

[3] Hazra NC , Rudisill C , Gulliford MC . Determinants of health care costs in the senior elderly: age, comorbidity, impairment, or proximity to death? Eur J Health Econ. 2018;19(6):831–42. Epub 2017 Aug 30. 28856487 10.1007/s10198-017-0926-2 PMC6008359

[4] Elderly population (indicator). OECD. 2019. Accessed 30 Jul 2021. 10.1787/8d805ea1-en

[5] The Japanese Registry of All Cardiac and Vascular Diseases (JROAD): Annual Report. 2016. Available from: http://www.j‐circ.or.jp/jittai_chosa/jittai_chosa2016web.pdf

[6] Dumbreck S , Flynn A , Nairn M , Wilson M , Treweek S , Mercer SW , et al. Drug‐disease and drug‐drug interactions: systematic examination of recommendations in 12 UK national clinical guidelines. BMJ. 2015;350:h949. 25762567 10.1136/bmj.h949 PMC4356453

[7] Mahoney FI , Barthel DW . Functional evaluation: The Barthel Index. Md State Med J. 1965;14:61–5. 14258950

[8] Charlson ME , Pompei P , Ales KL , MacKenzie CR . A new method of classifying prognostic comorbidity in longitudinal studies: development and validation. J Chronic Dis. 1987;40(5):373–83. 3558716 10.1016/0021-9681(87)90171-8

[9] Sweeney MO , Hellkamp AS , Ellenbogen KA . Adverse effect of ventricular pacing on heart failure and atrial fibrillation among patients with normal baseline QRS duration in a clinical trial of pacemaker therapy for sinus node dysfunction. Circulation. 2003;107(23):2932–7. Epub 2003 Jun 2. 12782566 10.1161/01.CIR.0000072769.17295.B1

[10] NICE.org [internet]. National Institute for Health and Care Excellence. Multimorbidity: clinical assessment and management. 2016 [cited 30 Jul 2021]. Available from: https://www.nice.org.uk/guidance/ng56/resources/multimorbidity‐clinical‐assessment‐and‐management‐pdf‐1837516654789

[11] Prazeres F , Santiago L . Measuring multimorbidity in family practice‐a comparison of two methods. Fam Pract. 2018;35(5):571–5. 29538672 10.1093/fampra/cmy014

[12] Prados‐Torres A , Calderón‐Larrañaga A , Hancco‐Saavedra J , Poblador‐Plou B , van den Akker M . Multimorbidity patterns: a systematic review. J Clin Epidemiol. 2014;67(3):254–66. 24472295 10.1016/j.jclinepi.2013.09.021

[13] Mori T , Hamada S , Yoshie S , Jeon B , Jin X , Takahashi H , et al. The associations of multimorbidity with the sum of annual medical and long‐term care expenditures in Japan. BMC Geriatr. 2019;19(1):69. 30841859 10.1186/s12877-019-1057-7 PMC6404301

[14] Jani BD , Hanlon P , Nicholl BI , McQueenie R , Gallacher KI , Lee D , et al. Relationship between multimorbidity, demographic factors and mortality: findings from the UK Biobank cohort. BMC Med. 2019;17(1):74. 30967141 10.1186/s12916-019-1305-x PMC6456941

[15] Kojima T , Akishita M , Nakamura T , Nomura K , Ogawa S , Iijima K , et al. Polypharmacy as a risk for fall occurrence in geriatric outpatients. Geriatr Gerontol Int. 2012;12(3):425–30. 22212467 10.1111/j.1447-0594.2011.00783.x

[16] Gutiérrez‐Valencia M , Izquierdo M , Cesari M , Casas‐Herrero Á , Inzitari M , Martínez‐Velilla N . The relationship between frailty and polypharmacy in older people: a systematic review. Br J Clin Pharmacol. 2018;84(7):1432–44. Epub 2018 May 3. 29575094 10.1111/bcp.13590 PMC6005607

[17] Lai SW , Liao KF , Liao CC , Muo CH , Liu CS , Sung FC . Polypharmacy correlates with increased risk for hip fracture in the elderly: a population‐based study. Medicine (Baltimore). 2010;89(5):295–9. 20827106 10.1097/MD.0b013e3181f15efc

[18] Sakamoto JI , Shikata T , Ito S , Kimura T , Takamoto K , Manabe E , et al. Polypharmacy is associated with accelerated deterioration of renal function in cardiovascular outpatients. Cardiol Res. 2020;11(1):15–21. 32095192 10.14740/cr991 PMC7011922

[19] Abe N , Kakamu T , Kumagai T , Hidaka T , Masuishi Y , Endo S , et al. Polypharmacy at admission prolongs length of hospitalization in gastrointestinal surgery patients. Geriatr Gerontol Int. 2020;20(11):1085–90. 32964583 10.1111/ggi.14044 PMC7756353

[20] Chen N , Alam AB , Lutsey PL , MacLehose RF , Claxton JS , Chen LY , et al. Polypharmacy, adverse outcomes, and treatment effectiveness in patients ≥75 with atrial fibrillation. J Am Heart Assoc. 2020;9(11):e015089. 32448024 10.1161/JAHA.119.015089 PMC7429010

[21] Masnoon N , Shakib S , Kalisch‐Ellett L , Caughey GE . What is polypharmacy? A systematic review of definitions. BMC Geriatr. 2017;17(1):230. 29017448 10.1186/s12877-017-0621-2 PMC5635569

[22] Kantor ED , Rehm CD , Haas JS , Chan AT , Giovannucci EL . Trends in prescription drug use among adults in the United States from 1999–2012. JAMA. 2015;314(17):1818–31. 26529160 10.1001/jama.2015.13766 PMC4752169

[23] Smith SM , Wallace E , O'Dowd T , Fortin M . Interventions for improving outcomes in patients with multimorbidity in primary care and community settings. Cochrane Database Syst Rev. 2021; 1(1): CD006560. 33448337 10.1002/14651858.CD006560.pub4 PMC8092473

[24] Wallace E , Salisbury C , Guthrie B , Lewis C , Fahey T , Smith SM . Managing patients with multimorbidity in primary care. BMJ. 2015;20(350):h176. 10.1136/bmj.h176 25646760

[25] Rea F , Biffi A , Ronco R , Franchi M , Cammarota S , Citarella A , et al. Cardiovascular outcomes and mortality associated with discontinuing statins in older patients receiving polypharmacy. JAMA Netw Open. 2021;4(6):e2113186. 34125221 10.1001/jamanetworkopen.2021.13186 PMC8204202

